# Non-Local Based Denoising Framework for In Vivo Contrast-Free Ultrasound Microvessel Imaging

**DOI:** 10.3390/s19020245

**Published:** 2019-01-10

**Authors:** Saba Adabi, Siavash Ghavami, Mostafa Fatemi, Azra Alizad

**Affiliations:** 1 Department of Radiology, Mayo Clinic College of Medicine & Science, Rochester, MN 55905, USA; adabi.saba@mayo.edu (S.A.); roudsari.seyed@mayo.edu (S.G.); 2 Department of Physiology and Biomedical Engineering, Mayo Clinic College of Medicine & Science, Rochester, MN 55905, USA; fatemi.mostafa@mayo.edu

**Keywords:** medical imaging, Doppler microvessel imaging, noise suppression, non-local based denoising, singular value decomposition

## Abstract

Vascular networks can provide invaluable information about tumor angiogenesis. Ultrafast Doppler imaging enables ultrasound to image microvessels by applying tissue clutter filtering methods on the spatio-temporal data obtained from plane-wave imaging. However, the resultant vessel images suffer from background noise that degrades image quality and restricts vessel visibilities. In this paper, we addressed microvessel visualization and the associated noise problem in the power Doppler images with the goal of achieving enhanced vessel-background separation. We proposed a combination of patch-based non-local mean filtering and top-hat morphological filtering to improve vessel outline and background noise suppression. We tested the proposed method on a flow phantom, as well as in vivo breast lesions, thyroid nodules, and pathologic liver from human subjects. The proposed non-local-based framework provided a remarkable gain of more than 15 dB, on average, in terms of contrast-to-noise and signal-to-noise ratios. In addition to improving visualization of microvessels, the proposed method provided high quality images suitable for microvessel morphology quantification that may be used for diagnostic applications.

## 1. Introduction

Imaging plays a key role in cancer screening, early diagnosis, and monitoring of disease progression. In addition to the pathological morphologic evidence of cancerous tissue changes, studying the associated vascular network and angiogenesis can provide valuable information on the nature of the tumor in order to improve the accuracy of cancer diagnosis [1]. For instance, early detection of breast cancer, the major cause of morbidity and mortality among women, is a prominent factor in survival rate [2]. Contrast-enhanced digital mammography [3]; contrast-enhanced magnetic resonance imaging [4]; diffuse optical imaging [5]; micro-computed tomography [6]; and, more recently, photoacoustic computed tomography [7] and contrast-enhanced ultrasound imaging [8] are the imaging techniques known to visualize the vascularity of the tumor, for breast cancer in particular. However, they are limited by the use of an exogenous contrast agent, radiation exposure, and cost burden. For contrast-enhanced ultrasound imaging, some advances on clutter signal suppression and, consequently, improving the vascular structure are reported in previous studies [9,10,11].

Ultrafast Doppler ultrasound (UFD) imaging can characterize the complex vascular network and flows because of its high framerate and large number of temporal samples [12], without the requirement for contrast agents. UFD offers, in order of magnitude, higher sensitivity of blood flow and microvasculature imaging compared with conventional Doppler ultrasound, as a result of plane-wave compounding and averaging of accumulated data over large time intervals [12]. The fact that the echo signal of moving blood cells has a larger Doppler shift than the echoes reflected from slowly moving tissue makes it possible to distinguish between those two signals either by using a high-pass filter [13,14] or principal component analysis-based adaptive filters [15]. Nonetheless, these filtering methods cannot effectively address the clutter removal problem without sacrificing small micro-vessel detection because of their static cut-off frequency. To mitigate these limitations, spatio-temporal filtering using singular value decomposition (SVD) was recently proposed to separate the two sub-spaces of blood flow and tissue via a thresholding operation [16,17,18,19,20]. In addition to the blood cells’ signature, there is a form of additive noise in the designated subspace due to the ultrasound system, in particular the time gain compensation circuit. The amplified additive background noise power and intensity fluctuations can change the intensity values and deteriorate vessel visibility, particularly in deeper regions of the image [20]. On the other hand, plane-wave imaging limits the penetration depth and accentuates background noise in the mid to deep regions of microvessel images when using global SVD filtering. The block-wise SVD is proposed to suppress the noise effectively by rejecting higher singular values, but it is computationally expensive [19]. The possibility of using a reference phantom in equalizing the background noise improves the visualization of vessel images without changing the signal-to-noise ratio (SNR) or contrast-to-noise ratio (CNR) [19]. Nevertheless, the reference phantom method is not adaptive and the gain cannot be well justified. In line with the same study, an adaptive method was presented to investigate the ramp-shaped background noise profile and derive the noise field from the lowest singular values and vectors obtained from SVD clutter filtering [21]. However, that method may introduce new artifacts to the vessel image. Recently, a method was presented to enhance vessel background separation on power Doppler images using an SVD-based technique and morphological top-hat filtering (THF) [20]. This method achieved a promising level of enhancement in terms of peak-to-side level gain; however, an additional level of denoising is needed to mitigate vast intensity fluctuations without affecting small vessel morphology, while preventing delineation of larger vessels. On the other hand, as explained by Vincent and Masters [22], it is cumbersome to perform mathematical morphological transforms, for example, THF, on images with large contrast variations. The non-local mean (NLM) image recovery paradigm offers a robust noise suppression and shows promise for enhancement of medical images [23]. The NLM technique is a graph Laplacian operation in patch intensity space that exploits self-similarities in images by comparing local neighborhoods [24,25]. The similarity between a given pixel pair is robustly derived from intensity differences between the patches of neighboring pixels surrounding them. Therefore, in this study, we used a combination of NLM filtering [23] and morphological THF to address background noise removal in ultrasound microvessel images. 

## 2. Materials and Methods

**Figure 1:**
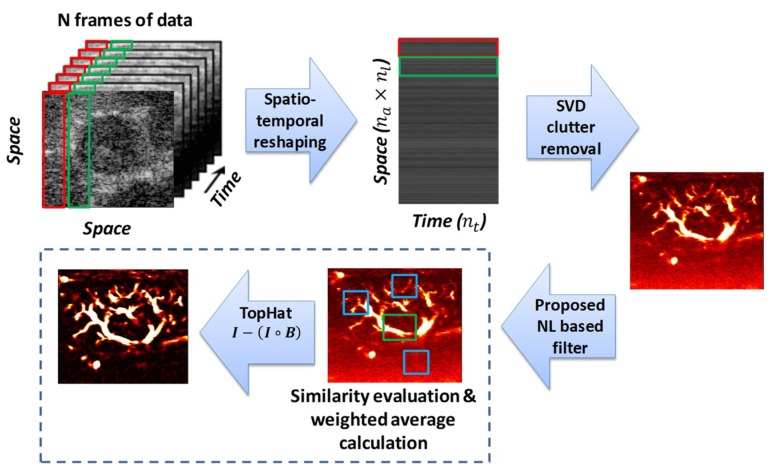
Schematic diagram of the proposed filtering framework. SVD—singular value decomposition.

Our proposed denoising framework for ultrasound Doppler microvessel image enhancement is summarized in Figure 1. The method is mainly based on NLM filtering. In particular, we used an SVD clutter filter first to obtain microvessel images. Then, we performed NLM-based [23] filtering, followed by a white THF to further suppress background noise. The evaluation was performed on a custom-made Doppler flow phantom and in vivo ultrasound images of malignant and benign breast lesions, thyroid nodules, and diseased liver. The study was approved by the Institutional Review Board (IRB) of Mayo Clinic, and was Health Insurance Portability and Accountability Act (HIPPA) compliant. A signed written informed consent was obtained from all participants prior to the study. The methods were carried out in accordance with the approved guidelines. Details of the methods are given in the following sections.

### 2.1. SVD Filtering and Clutter Rank Selection 

For rejecting the clutter signals from UFD datasets, we used a spatio-temporal method based on the SVD of data, employing the dissimilar spatial coherence of clutter and blood, as previously proposed [16,20]. Let the clutter and noise corrupted ultrasound signal be the following:

(1) $$r\left(x_{i,j},t_{k}\right)=\;r_{blood}\left(x_{i,j},t_{k}\right)+r_{clutter}\left(x_{i,j},t_{k}\right)+\upsilon \left(x_{i,j},t_{k}\right)$$

where$\;r_{blood}\left(x_{i,j},t_{k}\right),\;r_{clutter}\left(x_{i,j},t_{k}\right),\;$and $\upsilon \left(x_{i,j},t_{k}\right)$ are blood signal, clutter signal, and additive white Gaussian noise, respectively. Sampling locations are represented by the variable $x_{i,j}\;$ = **[**$\;x_{i},z_{j}]$ for lateral; ${\left(x_{i}\right)}_{i=1,..,n_{a}}$ and ${\left(z_{j}\right)}_{j=1,..,n_{l}}$ for axial; and ${\left(t_{k}\right)}_{k=1,..,n_{t}}$ for time. One can rearrange the ultrasound data to a two-dimensional space–time Casorati matrix of $R\left(x_{i,j},t_{k}\right)\in \;{\mathbb{R}}^{\left(n_{a}\times \;n_{l}\right)\times n_{t}}$. The SVD of such a matrix is written as Equation (2).

(2) $$R=\sum_{k=1}^{n_{t}}\lambda_{k}u_{k}v_{k}^{H}$$

where $u_{k}=\left[u_{1}\ldots u_{n_{a}.\;n_{l}}\right]$ and $v_{k}=\left[v_{1}\ldots v_{n_{t}}\right]$ are orthonormal bases, $\lambda_{k}$’s are singular values of the Casorati matrix, and H is the Hermitian operation. The clutter signal is approximated by a low rank matrix [16,17,19]. On the basis of this assumption, the tissue signal was concentrated on the first singular vectors of the Casorati matrix. Thus, suppression of the clutter signal can be accomplished by choosing an appropriate clutter rank. The rank, K, was selected based on setting a threshold on the slope of the second order derivative of the eigenvalues’ decay, as previously described [20]. Therefore, the reconstructed power Doppler intensity of red blood cells plus background noise is written as follows:

(3) $$R_{blood+noise,K}\left(i\right)=\sum_{k=K+1}^{n_{t}}\lambda_{k}^{2}\left|u_{k}^{2}\right|\left(i\right)\;i\in \left\{1,\ldots n_{a}\times \;n_{l}\right\},$$

To enhance the visibility and outlining of the microvessels, the background noise emerging in the designated blood cells’ subspace needed to be suppressed. We proposed a two-fold denoising framework, including an NLM filtering algorithm followed by morphological THF. 

### 2.2. Background Removal 

The main challenge of removing background noise in power Doppler images is preserving the integrity of small vessel structures. To address this challenge, we used a combination of a patch-based NLM filter, followed by a morphological-based THF on the power Doppler images. 

***Non-local mean filter***. The NLM denoising technique uses similar features (self-similarities) in images by comparing local neighborhoods. Mathematically, an image is called self-similar if any patches in the image can be approximated by other patches of the same image. The similarity between a given patch pair is derived using normalized L2-norm of the difference between those patches from neighboring patches surrounding them. Each given patch will be restored by a weighted average intensity of its similar patches in the image [26]. The patch-based NLM filtering method principally consists of dividing the image into patches with overlapping support and performing NLM filtering on these blocks. If a pixel is included in several blocks, various estimations of the same pixel from different NLM-filtered schemes are computed and stored to form the final restored intensity of pixels. Let the noisy ultrasound power Doppler image be u$\left(s_{i}\right)$ defined over a $\upsilon^{2}\in \mathbb{R}\;,\;$where $\upsilon^{2}$ is a rectangular bounded domain, $s_{i}$ is the intensity value, and *i* is the pixel index. Using a patch-based NLM filter recovery paradigm, as reported by Coupé [27], the restored block of pixels (***RB****_nl_*) $s_{i}$ is written as Equation (4a).

(4a) $$RB_{nl}\left(U\right)\left(\Pi_{i_{k}}\right)=\underset{\Pi_{j}\epsilon \;\varepsilon_{i_{k}}}{\sum }\xi \left(\Pi_{i_{k}},\Pi_{j}\right)u\left(\Pi_{j}\right)$$

(4b) $$\xi \left(\Pi_{i_{k}},\Pi_{j}\right)\;=\frac{1}{\zeta_{i_{k}}}\;\exp\left(\frac{-\sum_{p=1}^{P}{\left(U^{\left(p\right)}(\Pi_{i_{k}})-U^{\left(p\right)}(\Pi_{j})\right)}^{2}}{\eta }\right)\;$$

where $\Pi_{i_{k}}=\Pi \left(s_{i_{k}},\alpha \right)$ are the overlapping patches, that is, the partitions of the entire image centered on pixels $s_{i_{k}}$, and they contain $P={\left(2\alpha +1\right)}^{2}$ elements, α∈ℕ. Pixels $s_{i_{k}}$ are distributed equally at positions $i_{k}\;$=$\;(k_{1}n,k_{2}n)$; n is the distance between block centers;$\;u(\Pi_{i_{k}}$) =$\;[u^{\left(1\right)}(\Pi_{i_{k}}),\ldots ,u^{\left(p\right)}(\Pi_{i_{k}})){]}^{T}$ is the image patch gathering the intensity values of patch $\Pi_{i}$; $\varepsilon_{i_{k}}\;$is the square search centered at $s_{i_{k}}$ with size of ${\left(2M+1\right)}^{2}$ and M∈ℕ; $\xi \left(\Pi_{i_{k}},\Pi_{j}\right)$ is the weight used for restoring $U(\Pi_{i_{k}}$) given $U(\Pi_{j}$), and is calculated based on their similarity (L2-norm between two patches); η is the filtering parameter to regulate the decay of the exponential function in Equation (4b) and depends on the local neighborhoods; and $\zeta_{i_{k}}$ is the normalization constant that guarantees $\underset{\Pi_{j}\epsilon \;\varepsilon_{i_{k}}}{\sum }\xi \left(\Pi_{i_{k}},\Pi_{j}\right)=1$. For the pixel included in several patches, the computed multiple estimates are averaged to obtained the final restored image. 

***Top-hat morphological filter.*** THF is a morphological treatment for recognition of geometric features and is used for extracting intensity-dependent features in an image where a simple thresholding is not efficient [28]. A THF image, $I_{pth\;}$, is obtained by subtracting the opening of the NLM recovered image from itself as given in Equations (5a) and (5b).

(5a) $$I_{pth\;=}\;I_{pnl}-\left((I_{pnl}⊖B)⊕B\right)$$

and

(5b) $$I_{pnl}⊖B=\left\{x\;:{\;B}_{x}\subset I_{pnl}\right\}\;\mathrm{and}\;(I_{pnl}⊖B)⊕B\left\{x\;:{\;B}_{x}\cap^{\text{​}}(I_{pnl}⊖B)\neq \phi \right\}$$

where $I_{pnl}$ describes the gray-level image matrix resultant of NLM filtering, and B is the structural element matrix; and ⊕  and ⊖ show morphological dilation and erosion operations, respectively. THF acts like a high-pass filter and extracts the bright areas of the image, which are smaller than the mask. In this study, for the SVD method, the singular value decay acceleration threshold was optimized and set to 10-4 for best tissue clutter removal. The same value was used in all in vivo examples presented in this paper. The NLM and THF parameters, including smoothing criteria, were tuned to achieve the desired background removal while maintaining vessel intensity and delineation. 

### 2.3. Quantitative Assessment Measures 

In addition to visual assessment, we computed the regional SNR and CNR for power Doppler images as our quantitative assessment measures (QAM) in order to quantify the gain of the proposed method in comparison with applying an SVD filter or a combination of SVD and THF. The SNR and CNR are given by Equations (6) and (7), respectively, as follows:

(6) $$\mathrm{SNR}=20\log_{10}\left(\frac{{\bar{S}}_{bl}}{\sigma_{n}}\right)$$

(7) $$\mathrm{CNR}=20\log\left(\frac{{\bar{S}}_{bl}-{\bar{S}}_{clutter}}{\sigma_{n}}\right)$$

where ${\bar{S}}_{bl}$ is the mean of blood signal in the regions of interest (ROI), and $\sigma_{n}$ is the standard deviation of the background noise in the designated ROI. ${\bar{S}}_{clutter}\;$ is the mean of clutter signal in designated ROI. Three sets of different ROI were used in each image and the average values are reported throughout the paper. 

## 3. Results

The proposed algorithm was tested on a custom-made flow phantom and data set, as well as data from eight subjects with various abnormalities, that is, five breast lesions, which included benign breast fibroadenoma, malignant invasive ductal carcinoma (IDC) with grades II and III, metastatic renal cell carcinoma, two benign and malignant thyroid nodules, and two pathological liver subjects.

### 3.1. Validation of Flow Phantom Data 

To assess the performance of our method, a flow phantom was made using a small polycarbonate tube with an outer diameter of 850 µm (Paradigm Optics Inc., Vancouver, WA, USA) to transport the blood mimicking fluid. The surrounding environment was made from gelatin material using 300 Bloom gelatin and glycerol (Sigma-Aldrich, St. Louis, MO, USA) with a concentration of 5% by volume. A preservative of potassium sorbate (Sigma-Aldrich, St. Louis, MO, USA) was added with a concentration of 1% by volume. Cellulose particles (Sigma-Aldrich, St. Louis, MO, USA) with a size of 50 μm were also added with a concentration of 1% by volume to provide adequate ultrasonic scattering. The high frame rate plane-wave imaging was performed using a Verasonics programmable ultrasound machine (Verasonics, Kirkland, WA, USA) and a linear array transducer (L11-4, Philips, North America) at five-angle compounding $\left(-3^{\circ },-1^{\circ },0^{\circ },+1^{\circ },+3^{\circ }\right)$. A syringe pump (New Era Pump Systems, Inc., New York, NY, USA) was used to pump the blood mimicking fluid through the tubing. The system generated a sequence of 1000 frames in the form of raw in-phase/quadrature (IQ) beam-formed data with a duration of 2 s. The spatial resolution of the ultrasound data was 0.172 mm. Further processing was performed offline using MATLAB (Mathworks Inc., Natick, MA, USA). The maximum vessel size was chosen based on the SVD vessel image. The singular value decay acceleration was set to $10^{-4}$ for all cases. The top-hat (TH) structural element disk size was set to 13 pixels. The NLM parameters were tuned to achieve the best results and kept fixed for all experiments. The local neighborhood patch size was set to 3 × 3 pixels, and the search window size was 21 × 21 pixels, which were found to be robust for providing good results. The smoothing parameters were tuned to 0.01 given the amount of SNR and considering a functional approximation of noise variance. The ROI in the flow phantom were selected on the vessel area as well as in the background gelatin based region (green colored rectangles). 

**Figure 2:**
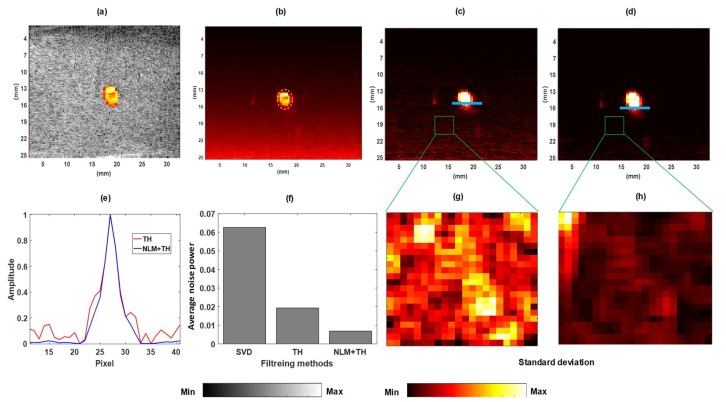
Flow phantom experiment results: (**a**) overlaid power Doppler image and B-mode; (**b**) unbiased singular value decomposition (SVD)-filtered image; (**c**) filtered image using SVD + top-hat (SVD+TH); (**d**) filtered image using proposed method non-local mean + TH (NLM+TH); (**e**) corresponding signal amplitude along blue lines in (**c**,**d**); (**f**) average noise power values for (**b**–**d**); and (**g**,**h**) are standard deviation maps for identified green color outlined background area in (**c**,**d**), respectively.

Figure 2 shows the visual comparison of (a) power Doppler image of the cross section of the flow tube overlaid the B-mode; (b) the calculated unbiased SVD image, which is obtained by removing the reconstructed image of the noise derived from the noise subspace [18]; (c) filtered vessel image using the TH filter; and (d) the filtered vessel image using the proposed method. The comparison shown in Figure 2e between the intensity signal along the identified blue line on image in (Figure 2c,d) shows the improvement of the proposed method (NLM+TH) over using SVD+TH. In Figure 2f, the average intensity values of background noise power for the three filtering methods are presented, and demonstrate that the average noise power in the proposed method has the minimum value. The calculation of the average of the background noise power was based on thresholding each image independently for noise and then exploiting the maximum value among three thresholds using Otsu’s method [29], which minimizes the intra-class variance. A similar analogy was applied to calculate the average vessel signal considering an empirical threshold. The standard deviation maps of the background region (outlined by the green color rectangle) illustrated in Figure 2g,h demonstrate that the background noise suppression with the proposed method outperformed the other two methods. 

Table 1 reports the SNR and CNR values for different filtering algorithms on the vessel image. The results show an incremental gain of about 12 dB in terms of SNR, and about 20 dB in terms of CNR, when comparing SVD-filtered images and images filtered with the proposed NLM-based method.

### 3.2. Evaluation Performance on In Vivo Clinical Data 

**Figure 3:**
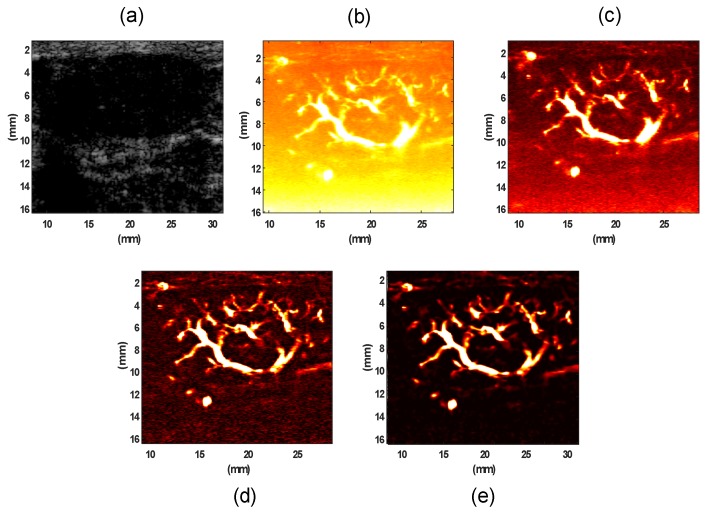
(**a**) B-mode image of a benign breast fibroadenoma (hypoechoic regions), (**b**) gross vasculature image using SVD, (**c**) vasculature image after unbiased SVD, (**d**) vasculature image using SVD + TH filtering (SVD+THF) filters, (**e**) final image of the vasculature after applying the proposed method (NLM+TH).

We also evaluated the performance of the proposed method on in vivo patient data. The data set was reconstructed using breast, thyroid, and liver conditions. The focus of study was mainly on the vessel structure within the tumor boundaries. We acquired all in vivo data using an Alpinion Ecube12-R ultrasound machine (ALPINION Medical Systems, Seoul, Korea). The L3-12H linear array (ALPINION Medical Systems, Seoul, Korea) with a centered imaging frequency of 8.5 MHz was used for studying subjects with thyroid nodules and breast lesions. Pathological liver imaging was performed using a curved array probe SC1-4H (ALPINION Medical Systems, Seoul, Korea) with the center frequency of 3.2 MHz. The system provided a sequence of frames at a high frame rate with five-angle compounded ultrasound raw data (at ~600 frames per second) in the form of raw IQ beam-formed data, for a total duration of 3.2 s on the lesion site. Figure 3 shows that the proposed method outperformed the other filtering methods in depicting vasculature images of a human breast lesion. 

**Figure 4:**
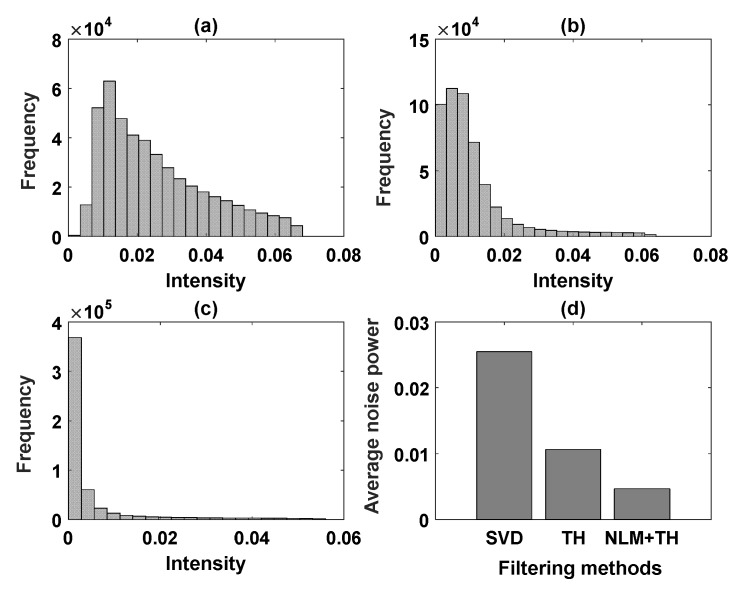
The intensity histogram comparison of noise level using (**a**) unbiased SVD, (**b**) SVD+TH, and (**c**) proposed method (NLM+TH); (**d**) average noise power for (**a**–**c**).

Similar to the phantom results, we calculated the average noise power using thresholding of each image for the noise model; then, we derived the maximum value among the three thresholds. A similar method was applied to calculate the average vessel signal. The histogram of background noise power and the average intensity value for the noise power calculated for unbiased SVD, SVD+TH, and the proposed method (NLM+TH) are compared in Figure 4. 

**Figure 5:**
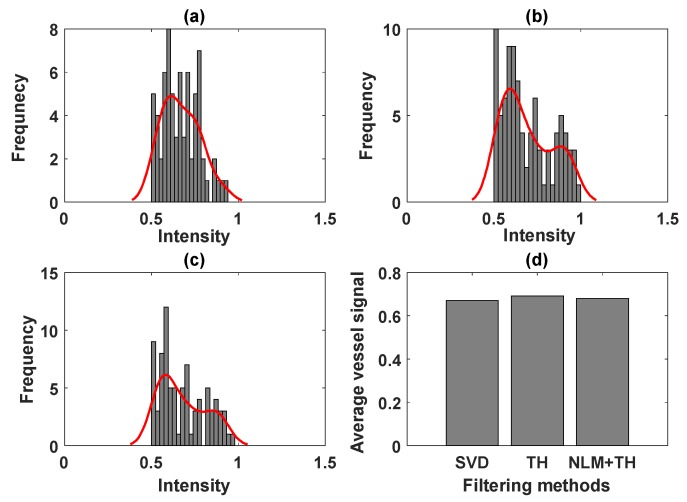
The intensity histogram comparison of vessel signal using (**a**) gross unbiased SVD, (**b**) SVD+TH, and (**c**) proposed method (NLM+TH); (**d**) average vessel signal of the gross unbiased SVD, SVD+TH, and proposed method (NLM+TH).

Comparing images in Figure 4a–c demonstrates that the mode of histogram shifted toward zero, an indication of noise power reduction. Figure 4d shows image improvement by the proposed method in terms of noise suppression compared with the other filtering methods. Moreover, we inspected the underlying vessel intensity distribution through the histograms for unbiased SVD, SVD+TH, and the proposed framework in Figure 5a–c, respectively. These results show that vessel intensity distributions remained relatively similar in all methods. The approximated average intensity values of vessel signal power were also calculated based on the same threshold value for the three filtering methods and are shown in Figure 5d. The plot in Figure 5d demonstrates that the proposed method maintained vessel signal strength, while noise was suppressed, as also shown in Figure 4d.

**Figure 6:**
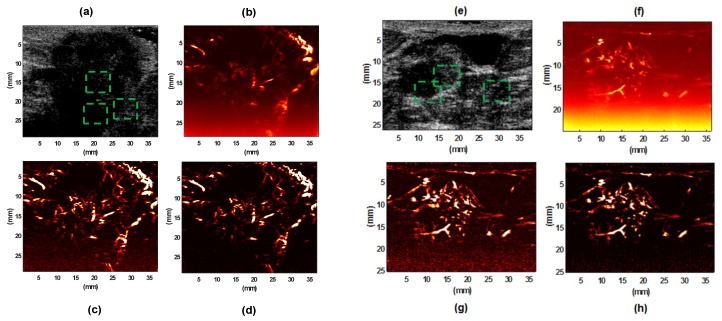
B-mode image of invasive ductal carcinoma (IDC) type III breast lesion (**a**) and IDC type II breast lesion (**e**); their gross vasculature SVD images (**b**,**f**), respectively; their vasculature images after SVD+TH (**c**,**g**), respectively; final images of vasculature after applying the proposed denoising method (NLM+TH) (**d**,**h**), respectively.

Extended examples of microvasculature images in human breast lesions for two IDC cases are given in Figure 6, which shows that the proposed method (NLM+TH) consistently outperformed the gross SVD vasculature and SVD+TH filters. 

By comparing the background noise in the resulting images, it becomes clear that the proposed restoration scheme efficiently preserved the high frequency components of the image corresponding to vessel structures, while removing the high frequencies due to noise. 

Our quantitative evaluation of the denoising method was based on regional SNR and CNR. Table 2 shows the SNR obtained for the three filtering methods for microvasculature images from five different breast lesions. Although these images have slightly different levels of noise, our proposed method (NLM+TH) produced the best SNR with an average incremental gain of 18 dB. The CNR of ROI for the same five breast cases using the three methods are given in Table 3. On average, an improvement of 10 dB was observed for CNR using the proposed framework compared with using SVD or SVD+TH. 

**Figure 7:**
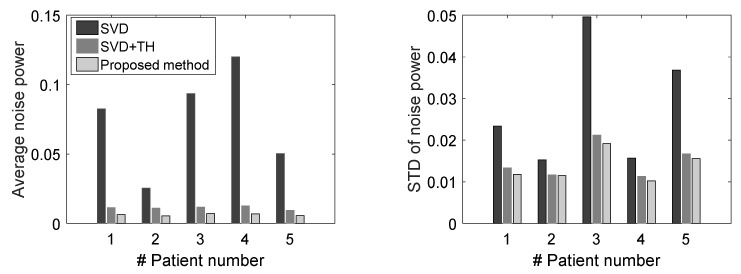
Mean and standard deviation of noise power for the microvasculature images of five breast lesions, comparing SDV, SVD+TH, and the proposed method (NLM+TH).

The overall results show the advantages of the proposed framework in preserving fine details, while suppressing strong background noise. To reach a more general conclusion, we also looked into the noise power mean (Figure 7, left) and standard deviation (Figure 7, right) for the five breast lesions using the three filtering methods on power Doppler images. The results clearly showed that the average noise power, calculated based on thresholding in Section 3.1, was lower when using the proposed NLM-based filter compared with using solely SVD or SVD+TH filtering. 

**Figure 8:**
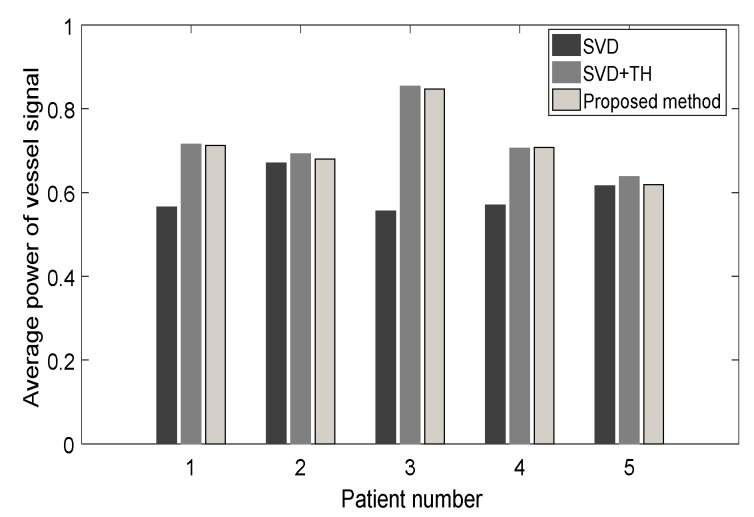
Average vessel power signal for microvasculature images of five breast lesions, comparing SDV, SVD+TH, and the proposed method (NLM+TH).

In terms of standard deviation of noise power, there was a promising improvement for the proposed method compared with SVD alone and SVD+TH filtered microvasculature images. Moreover, the vessel power signature was improved using the proposed method in all cases compared with the gross SVD microvessel images (see Figure 8). 

**Figure 9:**
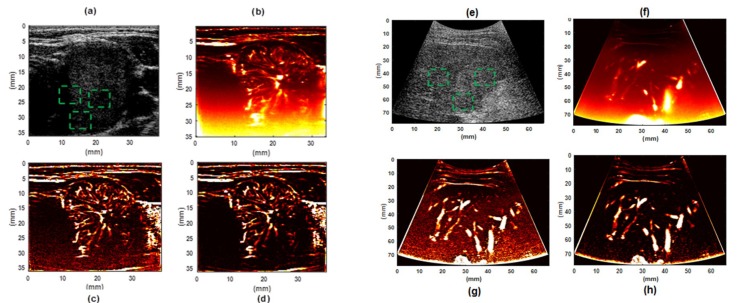
B-mode images of a (**a**) malignant thyroid nodule and (**e**) a pathological liver; (**b**,**f**) are their gross vasculature SVD images; (**c**,**g**) are their vasculature images after SVD+TH, respectively; and (**d**,**h**) are the final images of vasculature after applying the proposed denoising method (NLM+TH).

To demonstrate the quantitative and qualitative gain of the proposed filtering framework in imaging other organs, representative cases from liver and thyroid are presented in Figure 9 left and right panels, respectively. The proposed method (NLM+TH) provided a significant improvement in microvessel visualization in both organs. Moreover, the level of enhancement in liver cases validated using a different probe setup and did not affect the outperformance of the proposed denoising method.

The quantitative evaluation of thyroid and liver cases is given in Table 4 and Table 5. As can be observed, the proposed method provided a substantial improvement of about 10 dB on average in terms of SNR and CNR for both thyroid and liver cases, while offering better vessel visualization compared with the unbiased SVD vasculature images.

## 4. Discussion

Non-contrast vascular imaging using high frame rate plane-wave imaging and tissue clutter removal techniques can play a major role in visualization of neovascularization in tumors. However, the additional background noise that remains after clutter filtering can obstruct the small vessels and degrade image quality. The overall objective of our study was to develop a framework for suppressing the background noise and to assess the quantitative and qualitative merits of images obtained by this framework relative to those obtained by SVD filtering, as well as a combination of SVD followed by the morphological THF. We devised a combination of NLM and morphological THF to mitigate vast intensity fluctuations, while maintaining the morphological integrity of small vessels and preserving lager vessels. The proposed filtering technique enabled equalization and suppression of background noise by reducing the average and standard deviation of noise power, respectively. In contrast to the local kernel regression method, the NLM method breaks the locality constraint in conventional restoration methods and estimates the pixel value from all similar patches collected from a large region. In a non-local recovery paradigm, a pixel-based NLM filter replaces a noisy pixel by the weighted average of other image pixels, where weights reflect the similarity between the local neighborhood of the pixel being processed and those of other pixels. It takes advantage of the redundancy of similar patches existing in the target image for the denoising task. A patch-based implementation of NLM for noise reduction has been introduced to overcome the computational complexity of pixel-based NLM [30]. One of the main difficulties in achieving the desired NLM-based filter in our study was tuning the filter parameters, including a smoothing parameter and selection of the patches. In the similarity evaluation and weighting process, the normalized L2-norm was computed between two patches, as reported by Fukumura and colleagues [1]. The standard deviation of the Gaussian kernel was used to assign spatial weights to the patch. To avoid useless weight computations, the method proposed by Mahmoudi and Sapiro [31] was used to preselect a subset of the most relevant patches in the search window. 

One hurdle for the experimental dataset was the fact that because the ground truth was unknown, we calculated regional quality assessment measures and compared the filtering methods in terms of preserving high intensity features and low intensity uptakes. In spite of compelling results, the outcome of the NLM filters cannot be absolutely optimal because of the imperfect estimation of the noise standard deviation in the image. To improve upon these results, a further investigation should be conducted based on using Bayesian estimation NLM, which can more precisely estimate and fine tune filter parameters. Moreover, it is important to underline that in the top part of power Doppler images, the regional QAM values for the proposed method and the SVD+TH filtered image are relatively similar; however, noise suppression is more pronounced in deeper areas of the image. To obtain an optimum filter, the denoised image needs to balance edge preservation and noise removal. We showed pathological cases in which the filter preserves the major visual signature of the given pathologic angiogenesis. Finally, the impact of the NLM-based denoising on the performances of post processing algorithms, like the vessel segmentation scheme, should also be studied in greater detail. The preliminary qualitative and quantitative results presented here suggest that the proposed patch-based NLM filter framework can successfully improve microvessel images. 

## 5. Conclusions

We introduced an NLM-based denoising framework for in vivo contrast-free ultrasound microvessel images. The algorithm provided a two-fold enhancement of ultrasound microvessel images using a non-local based restoration and a morphological THF. Evaluations were performed on in vivo breast, thyroid, and liver microvascular images. The findings demonstrated that the proposed filtering method outperformed the gross SVD filtering, as well as SVD followed by THF. A visual and quantitative assessment showed the proposed method offers significant gain in terms of vessel outlining that potentially facilitates vessel morphologic quantifications [32] that may be used for diagnostic applications in the clinic. 

## Figures and Tables

**Table 1:** Quantitative assessment measures (QAM) calculation for flow phantom. SNR—signal-to-noise ratio; CNR—contrast-to-noise ratio; SVD—singular value decomposition; TH—top-hat.

QAM (dB)	SVD	SVD+TH	Proposed
SNR	30.80	32.21	42.07
CNR	27.23	31.12	41.52

**Table 2:** SNR values in dB obtained by applying three different filtering methods on microvasculature images of five breast lesions.

Breast Lesion#	SVD-Unbiased	SVD+TH	Proposed
1	32.77	31.07	40.20
2	29.16	34.44	51.45
3	27.96	34.26	45.25
4	22.13	25.21	35.34
5	39.91	32.93	38.38

**Table 3:** CNR values in dB obtained by applying three different filtering methods on microvasculature images of five breast lesions.

Breast Lesion#	SVD-Unbiased	SVD+TH	Proposed
1	27.88	30.57	38.72
2	27.49	32.90	42.69
3	32.71	31.60	42.20
4	23.49	29.20	38.49
5	29.98	33.49	41.01

**Table 4:** SNR values (in dB) of microvessel images of two thyroid nodules and two liver cases using three different filtering methods.

Organ	SVD-Unbiased	SVD+TH	Proposed
Thyroid 1	29.39	32.41	38.79
Thyroid 2	34.75	40.77	45.94
Liver 1	33.87	38.06	45.23
Liver 2	20.01	25.61	31.26

**Table 5:** CNR values (in dB) of microvessel images of two thyroid nodules and two liver cases using three different filtering methods.

Organ	SVD-Unbiased	SVD+TH	Proposed
Thyroid 1	24.39	29.74	32.70
Thyroid 2	31.26	38.96	42.20
Liver 1	28.49	37.61	45.26
Liver 2	20.01	25.61	31.26
